# Questionable Immunity to Mumps among Healthcare Workers in Italy—A Cross-Sectional Serological Study

**DOI:** 10.3390/vaccines12050522

**Published:** 2024-05-10

**Authors:** Cristiana Ferrari, Giuseppina Somma, Michele Treglia, Margherita Pallocci, Pierluigi Passalacqua, Luca Di Giampaolo, Luca Coppeta

**Affiliations:** 1 PhD Program in Social, Occupational and Medico-Legal Sciences, Department of Occupational Medicine, University of Rome Tor Vergata, Viale Oxford 81, 00133 Roma, Italy; 2 Department of Occupational Medicine, University of Rome Tor Vergata, Viale Oxford 81, 00133 Roma, Italy; giuseppina.somma@ptvonline.it (G.S.); michele.treglia@uniroma2.it (M.T.); luca.coppeta@uniroma2.it (L.C.); 3 PhD Program in Applied Medical Surgical Sciences, Department of Surgical Sciences, University of Rome Tor Vergata, Viale Oxford 81, 00133 Roma, Italy; margherita.pallocci@gmail.com; 4 Department of Occupational Medicine, University of Rome La Sapienza, 00185 Roma, Italy; p.passalacqua92@gmail.com; 5 Department of Occupational Medicine, University of Chieti “G. D’Annunzio”, 66100 Chieti, Italy; l.digiampaolo@unich.it

**Keywords:** mumps, healthcare workers, immunity, vaccines, MMR vaccine

## Abstract

Highly contagious diseases, such as mumps, are a global concern as new epidemics continue to emerge, even in highly vaccinated populations. The risk of transmission and spread of these viruses is even higher for individuals who are more likely to be exposed, including healthcare workers (HCWs). In healthcare settings, both HCWs and patients are at risk of infection during the care process, potentially leading to nosocomial epidemic outbreaks. Mumps is often underestimated compared with measles and rubella, despite being milder and less likely to spread. In fact, the risk of complications following mumps infection is extremely high, especially if the disease occurs in adulthood. The measles–mumps–rubella (MMR) vaccine has been shown to be an excellent preventive measure. Unfortunately, the mumps component appears to be less effective in inducing immunity than those for measles and rubella (two-dose effectiveness of 85%, 95% and 97%, respectively). The main aim of our study was to investigate the prevalence of detectable mumps antibodies (serum IgG antibodies) in a cohort of Italian and foreign HCWs in relation to personal and occupational factors. We included in the study 468 subjects who underwent health surveillance at the Occupational Medicine Unit of the Tor Vergata Polyclinic in Rome during the period from January 2021 to March 2023. In our study, the proportion of HCWs found to be unprotected against mumps was very high (8.3%), and those found to be immune are below the WHO threshold for herd immunity (95%). From our data, it seems essential that all occupational health services carry out an accurate screening with a dose of anti-mumps antibodies to assess serological protection before starting a job, regardless of an individual’s vaccination history. This approach is proving to be beneficial, accurate, as it allows all serologically non-immune individuals to be vaccinated in the workplace, including those who would be protected by their vaccination history but have lost the antibody response.

## 1. Introduction

Healthcare workers are believed to be at a higher risk of contracting mumps compared to the general population [1,2,3]. Healthcare-related mumps is a public health concern because of the potential for non-immune healthcare workers to infect susceptible patients and colleagues, and mumps disease can spread very rapidly in hospital settings [1,4].

Compared with measles and rubella, the mumps vaccine is less immunogenic; therefore, it is possible that a considerable number of HCWs may fail to develop protective antibody titers when vaccinated, also following a full MMR vaccination (two doses). The protective threshold of mumps IgG is unknown, but it is recognized that individuals with low antibody levels can become infected if exposed to the mumps virus [5,6,7].

The Italian National Vaccination Plan recommends that one dose of MMR vaccine be given to HCWs who do not have a complete vaccine history or who test seronegative for at least one of the three pathogens [8].

For mumps infection, the Advisory Committee on Immunization Practices (ACIP) has recommended an additional dose of mumps vaccine for HCWs, previously vaccinated with two doses of MMR vaccine, and at higher risk of mumps infection in case of an epidemic [9]. However, in Italy, the recommended vaccination schedule for MMR consists of two doses. This may pose a possible risk, as vaccinated workers may not be protected against mumps [3].

In 2022, the Italian Society of Occupational Medicine (SIML) published “Operational guidelines on the main vaccinations for healthcare workers”, which include the MMR vaccination with a schedule of two doses at least four weeks apart for measles and mumps, and at least one dose for rubella. However, they do not recommend a booster dose for those who have previously had a complete cycle of vaccination and are serologically negative, unless they are at high risk due to mumps epidemics, in which case they should be given a third booster dose of MMR vaccine [10].

Recent outbreaks of mumps in individuals who had received two doses of MMR vaccine have called into question the effectiveness of the vaccine. However, clinical symptoms, complications, viral shedding and transmission associated with mumps infection have been shown to be reduced in vaccinated individuals, demonstrating a benefit of this vaccine. It is also important to evaluate the number of cases in recent mumps outbreaks to determine whether this is a real increase or whether the improved ability to detect acute infection is a contributory factor [11].

The exact nature of the immune response to the mumps vaccination strain is unknown. However, several young adults immunized at childhood in the past twenty years have shown a higher risk of mumps infection over the years, and it is believed that this is associated with a decrease in antibody levels below the protective threshold [12,13,14].

Possible reasons for inadequate immune protection include primary vaccine failure, secondary vaccine failure, or waning immunity.

Primary vaccine failure is the absence of a protective immune response due to a lack of sufficient initial antibody response to a vaccine in a host [15,16]. This type of vaccine failure may be caused by improper storage, manipulation, or administration of the vaccine, which can affect its efficacy. Alternatively, it may be due to an ineffective initial immunological response in an individual. Although there are few follow-up studies to estimate the antibody titers specific to the mumps vaccine strain after vaccination, the evidence from several published papers suggests that primary vaccine failure is not a significant determinant of the epidemics that have occurred [17,18,19].

Secondary vaccine failure is the progressive loss of immunity after an early positive response, occurring over several years after immunization.

The waning of immunity is a decrease in immune protection proportional to time since immunization. A possible decline in immunity has been documented in the recent mumps epidemics in Europe and the United States, which mainly concern young adults from well-vaccinated groups who are in university education and who were vaccinated with two doses of MMR vaccine in infancy [12,20,21,22].

According to a recent meta-analysis that used a mathematical approach, there is a decline in immunity against mumps approximately 27 years after vaccination [23], while another study showed an approximately 20% decline in mumps-neutralizing antibody levels over one decade [24]. However, some research contradicts these conclusions, demonstrating that there is no link between protection against mumps and the period since vaccination [25,26,27]. Some authors have found that 70–99% of people showed detectable mumps titers approximately 10 years after the first dose of vaccine [28,29]. Another study showed minimal decline in antibody levels after two doses of MMR vaccine, 6–7 years after the second vaccination [30].

Administering an additional dose of the MMR vaccine has been proven to represent a successful stopgap measure to limit the spread of the virus during epidemics [31,32,33,34].

It is yet to be proven if the complete absence of measurable antibodies is associated with a decline in clinical defense, as the threshold level of neutralizing antibodies necessary for immunity to mumps has not yet been identified [26].

Subjects without detectable levels of mumps-specific antibodies could become infected but remain protected from severe disease because they may be shielded by cell-mediated immune memory, with T and B cell immunity lasting up to 10 years after vaccination [35,36].

The importance and effectiveness of T-cell immunity in mumps infections is unclear. However, it is possible that some strains of mumps virus are able to escape T cell responses induced by vaccination, which cannot not be evaluated as significant until B cell immunity wanes [37].

The aim of our research was to investigate the prevalence of immunity to mumps by the evaluation of the serological status in a cohort of Italian and foreign HCWs in relation to socio-demographic and occupational factors.

## 2. Materials and Methods

We performed a retrospective analysis of the immunological status, by evaluating the antibody titers against mumps and collecting information on the vaccination status, of all healthcare workers who underwent health surveillance at the Occupational Medicine Unit of the Tor Vergata Polyclinic (PTV) in Rome during the period from January 2021 to March 2023.

All individuals included in the research sample underwent routine venous blood sampling, including titration of MMR-specific IgG antibody levels. The VirClia^®^ Lotus method, an indirect chemiluminescent immunoassay (CLIA), was used to test for antibodies to the mumps virus in human serum/plasma. We considered a mumps-specific IgG level above 1.1 S/CO (signal to cut-off ratio) to be protective, according to the manufacturer’s specifications.

Quantitative data were expressed as mean ± SD, and categorical variables were expressed as number and percentage of subjects. In the statistical analysis, the Chi-square test was used to evaluate the association between serological protection and the main characteristics of the population (gender, age, work occupation, country of origin, and years of service). Results were considered statistically significant if the *p*-value was <0.05.

Statistical analysis was performed using IBM SPSS software (version 26).

## 3. Results

### 3.1. Study Population

The study population consisted of 468 subjects, 304 females (65.0%) and 164 males (35.0%), with a mean age of 32.99 ± 10.03 years (range 22–68). In particular, females had a mean age of 32.20 ± 9.24 years, while males had a mean age of 34.46 ± 11.23 years (*p* = n.s.). Regarding the tasks of the subjects studied: 202 were physicians, 136 were nurses, 96 were technicians, and 34 were other health professionals (psychologists, social workers, speech therapists, etc.).

In terms of country of origin, 418 were Italian subjects and 50 were from other countries.

Regarding the continental distribution, only one participant was from Central America, one was from North America, two were from South America, and eight were from Asia. Most of the participants (97.5%) were from Europe (456/468), of which 91.7% were from Italy (418/456).

As the majority of subjects were unable to provide a documented history of MMR vaccination, this information was excluded from further analysis.

### 3.2. Serological Evaluation

A detectable antibody level was found in 91.7% of the subjects (429/468), while 39 were subjects lacking protective antibody titers.

The mean antibody titer was 3.17 S/CO ± 1.27 (range 0.12–8.69).

Of the men in the study, 92.7% were protected (152/164), whereas only 91.1% of the women in the study population were protected (277/304). This gender difference was not statistically significant in the Chi-square test with a *p*-value = 0.35.

We examined possible differences in antibody levels according to the tasks of the subjects studied, which could reflect the different types of exposure to the virus. In general, nurses are considered to be a higher-risk population; 90.4% of nurses (123/136), 93.1% of physicians (188/202), 91.7% of technicians (88/96), and 88.2% of other professions (30/34) were protected. These differences in antibody levels in the different occupational categories, although evident, were not statistically significant. The mean titer for the different tasks was 3.05 ± 1.27 S/CO for nurses, 3.30 ± 1.30 S/CO for physicians, 3.03 ± 1.20 S/CO for technicians, and 3.33 ± 1.25 S/CO for other personnel (*p* = 0.94).

Since we assumed that the percentage of vaccinated persons born before 1990 was low and that serological immunity in these subjects was due to natural infection, we evaluated the percentage of immune workers in the oldest category of our sample (> 50 years) on the basis of historical data on the circulation of mumps in Italy. In these subjects, both the rate of subjects with a detectable antibody level and the titer were higher than in the other group (serological protection: 95.3% vs. 90.3%; *p* = n.s.; mean titer: 3.30 S/CO vs. 3.08 S/CO; *p* < 0.05).

All results regarding the main characteristics of the study population (gender, occupational task, years of service, age class and continent) and the associated protection rates and mean antibody titers are shown in Table 1 and Table 2.

## 4. Discussion

The COVID-19 pandemic could be playing a significant part in lowering the incidence of respiratory infections such as mumps worldwide in 2021 [38,39,40]. In fact, the containment activities carried out throughout the pandemic, like stay-at-home policies, school closures, reduction in social contacts, use of respiratory protective equipment, and adoption of more stringent hygiene measures, may have helped to reduce the transmission of several respiratory diseases, including mumps. The additional workload for public health services may have led to underreporting of mumps cases by both physicians and public health workers; therefore, reported epidemiological data should be interpreted with caution. The peak in the reporting rate of mumps cases observed in 2019 (overall EU/EEA rate 4.2) was mainly influenced by a large, long-lasting outbreak reported from Ireland. The outbreak began in the second half of 2019 and continued to generate cases until early 2020, mainly affecting adolescents and young adults. The main factors contributing to this outbreak were reported to be the crowded social environment of students, historically low uptake of MMR vaccine, insufficient efficacy of the mumps component of the MMR vaccine, and the possibility of waning immunity in vaccinated individuals. This outbreak confirms that mumps cases can still emerge in the EU/EEA, and it is therefore of paramount importance to achieve large vaccination coverage with at least two doses of MMR vaccine in all children and young adults. Similar epidemics have been widely reported in the literature, with a high percentage of cases in completely immunized individuals [41,42,43,44].

EU/EEA data between 2017 and 2021 showed that 40% of mumps cases in individuals with known vaccination status had received at least two doses, and there was a significant overrepresentation of individuals aged 10–29 years. This may be due to a combination of incomplete protection from the mumps component of the MMR vaccine, waning immunity, or close social interactions that may make the virus easier to transmit [41,42].

The latest WHO data show that overall estimates of vaccination coverage in the EU/EEA have remained largely unchanged over the past four years. During the pandemic, a strong weakness of national immunization programs was observed worldwide, with a significant decrease in coverage in the first quarter of 2020. A striking example is Iceland, where coverage of the second dose of the measles-containing vaccine fell from 95% in 2018 to 10% in 2021. Otherwise, vaccination programs in the EU/EEA seemed to be more resistant. Many countries succeed in achieving coverage in 2020 and 2021 similar to that of 2018 and 2019 [45].

Although MMR vaccination in infancy may not provide total individual immunization against mumps in later life, the relevance of keeping high MMR vaccination coverage cannot be underestimated. The vaccine has been shown to be highly effective in reducing overall morbidity and mortality from all three viruses it protects against [46]; high vaccination coverage reduces the possibility of epidemics in the general population [7,47], and vaccination also has a direct protective effect on the severity of mumps symptoms [48,49]. An additional dose of MMR vaccine may be successful in reducing the risk of mumps in the case of an epidemic [34], but the low duration of the antibody response seen after a third dose of vaccine has generated concerns about its wider applicability beyond epidemic control [50,51].

The results of our study show no significant gender differences in protection against mumps, data that are fully consistent with those reported in the literature [52]. Women usually develop a stronger immune response (both humoral and cell-mediated) and more severe side effects after vaccination. The adverse effects can be explained by the greater activation of the immune system due to the presence of polymorphisms and variability in the genes of the sex chromosomes; in addition, hormonal differences may influence this humoral immune response in the autosomal genes coding for immunological proteins. On the contrary, the clinical manifestations and course of the disease are more severe in men, who also have a higher incidence of complications [53].

The healthcare sector represents a high-risk area for the spread of mumps, despite the presence of a highly vaccinated population, as evidenced by several epidemic cases documented in the literature [4,47,54,55].

Subjects under 23 years of age are the population vaccinated by compulsory vaccination (Legislative Decree 73/2017) and therefore represent a highly protected group (93.8%), albeit with a lower antibody titer (2.87 ± 1.16S/CO, vs. 3.20 ± 1.29 of subjects aged > 23) [56]. The vaccination coverage rate for measles, mumps and rubella in Italy in 2021 was approximately 93.8% of children. This level of coverage is below the optimal threshold, as indicated by the WHO, which suggests that almost 95% of children should be vaccinated to achieve herd immunity [57].

However, subjects over 50 years of age represent a group with a higher protection rate (95.3% vs. 90.3%), most likely due to a previous natural infection, in historical periods when the virus circulation was higher and the hygienic–sanitary conditions in the places most at risk of infection were worse than today. The natural infection also gave them a higher average antibody titer than the other age groups (3.30 S/CO vs. 3.08 S/CO) [57,58,59].

In summary, vaccination provides higher protection rates but results in a relatively lower antibody titer. In contrast, natural immunity results in more durable immune protection and a greater antibody response, and it has also been shown that post-infection antibodies have a half-life of 50 years or more [58,59,60].

However, the percentage of people who were unprotected against mumps was still too high (8.3%) when all the categories of people studied were taken into account. These data are particularly worrying because they mainly concern the most vulnerable groups, i.e., nurses, whose work brings them into close contact with patients.

The vaccination rate in the medical population is higher than in any other occupational group. One of the reasons for this result is undoubtedly the greater general acceptance of having a booster, if necessary, given the lower level of vaccine hesitancy in this category. In fact, according to the literature, doctors are a group that has greater confidence in vaccination, in contrast to nurses, who seem to be the subjects who express higher levels of vaccine hesitancy towards the different categories of vaccines available [61,62].

In addition, doctors themselves have less close and frequent contact with patients than nurses, but they are more likely to be the first to encounter a person with mumps, as a patient with mumps is usually someone who is not admitted to hospital but who has a medical diagnosis without going to hospital.

There were no statistically significant differences in protection rates according to the continent of origin of the subjects included in the study, although the sample was not large enough to represent all countries with a comparable number of subjects. However, it would be useful to look more closely at the vaccination guidelines and indications followed and implemented in the different countries, both in childhood and in specific risk categories such as HCWs.

As with measles and rubella, serological screening allows occupational health practitioners to avoid inappropriate administration of vaccine to individuals who have been previously vaccinated and are protected but cannot provide proof of vaccination [63]. Therefore, the assessment of mumps IgG levels can be considered more accurate than assessing reported vaccination history, allowing the correct identification of the immunological status of HCWs, regardless of a previous vaccination cycle (complete or incomplete). At present, individuals who have previously received two doses of MMR vaccine are considered immune and are not tested for antibody titers. This is based on current recommendations that a third dose should not be given, except to those working in contexts where there is a risk of contact with sick people in the event of an outbreak. The persistence of immune memory (both humoral and cell-mediated) for mumps has not been definitively established, and we cannot exclude the possibility that these workers may in fact be susceptible if exposed to a contagious case. However, the method of pre-vaccination anamnestic screening proposed in the current recommendations (which does not include mandatory serological testing) could lead to individuals with low antibody titers being considered “immune” because they have been vaccinated.

In conclusion, the current definition of persons to be vaccinated according to recent recommendations, based solely on a positive vaccination history, is inaccurate and should be discouraged as a screening method. Therefore, recommendations for vaccination of HCWs against mumps should be based on the prior determination of the antibody titer together with the vaccination history in non-immune individuals.

Our study has some limitations. First, the study population was not divided into homogeneous subgroups but according to the department to which they were assigned. It is known that the risk of exposure to mumps is not uniform throughout the hospital, as some departments are more dangerous because of the type of patients they treat (e.g., emergency departments, infectious diseases, pediatrics, intensive care, oncology, hematology). A second limitation of our study is its hospital-based nature, involving 468 individuals with unclear vaccination histories and reliance on a single blood test. Consequently, the roles of vaccination and natural infection remain ambiguous. As 50 operators included in our study came from abroad, it would have been interesting to delve deeper and consider the different vaccination calendars followed in each country to discuss the differences found in more detail. Another limitation of our study is that we did not take into account the type of vaccine received at the time of the vaccination history, as there may be differences in antibody response and protection rates related to the type of vaccine used.

## 5. Conclusions

Airborne infectious diseases such as measles, rubella and mumps remain a significant global problem, with new epidemics occurring frequently even in highly vaccinated populations.

The risk of transmission and spread of these viruses is even greater for those categories of people who are more likely to be exposed, such as healthcare workers.

The proportion of HCWS (doctors, nurses, technicians and other workers) who were still unprotected against mumps in our study is very high (8.3%), and those who are immune are below the WHO threshold for herd immunity (95%).

It is therefore essential for all occupational health services to carry out accurate screening for anti-mumps antibodies prior to commencing work or training to assess serological protection, regardless of the vaccination history of the individual who is not serologically immune, including those who would be protected by vaccination history but have lost the antibody response.

In conclusion, contrary to current recommendations and guidelines, it appears necessary to perform serological screening and offer a booster dose of MMR vaccine to all workers and students who are serologically unprotected and at increased risk of infection, regardless of their vaccination status.

## Figures and Tables

**Table 1:** Main characteristics of the study population.

Categories		Number	Percent (%)
**Gender**	Male	164	35
	Female	304	65
**Task**	Nurses	136	29.1
	Physicians	202	43.2
	Technicians	96	20.5
	Other	34	7.2
**Years of service**	<10	229	53.8
	>10	197	46.2
**Continent**	South America	2	0.4
	Central America	1	0.2
	North America	1	0.2
	Asia	8	1.7
	Europe	456	97.5
	Italy	418	89.3 (91.7 of European)
**Age class**	>50 years	40	8.5
	≤50 years	428	91.5
	>23 years	404	86.3
	≤23 years	64	13.7

**Table 2:** Protection rates and mean antibody titers of study population.

Categories		Protected (%)		Mean Titer		*p*-Value
		YES	NO	N (S/CO)	±sd	
**Gender**	Male	92.7	7.3	2.99	1.38	n.s.
	Female	91.1	8.9	3.13	1.26	
**Task**	Nurses	90.4	9.6	3.05	1.27	n.s.
	Physicians	93.1	6.9	3.30	1.30	
	Technicians	91.7	8.3	3.03	1.20	
	Other	88.2	11.8	3.33	1.25	
**Year of** **service**	<10	92.6	7.4	3.08	1.25	n.s.
	>10	90.9	9.1	3.24	1.31	
**Continent**	South America	50.0	50.0	3.19	1.43	n.s.
	Central America	100	0	3.59	0.28	
	North America	100	0	3.51	1.40	
	Asia	87.5	13.5	3.09	1.41	
	Europe	91.8	8.2	3.18	1.27	
**Age class**	>50 years	95.3	4.7	3.30	1.09	<0.05
	≤50 years	90.3	9.7	3.08	1.29	
	>23 years	91.3	8.7	3.20	1.29	n.s.
	≤23 years	93.8	6.3	2.87	1.16	

## Data Availability

The data presented in this study are available on request from the corresponding author.

## References

[1] Fortunato F., Tafuri S., Cozza V., Martinelli D., Prato R. (2015). Low vaccination coverage among Italian healthcare workers in 2013. Hum. Vaccines Immunother..

[2] Coppeta L., Balbi O., Baldi S., Pietroiusti A., Magrini A. (2019). Pre-vaccination IgG screening for mumps is the most cost-effectiveness immunization strategy among Health Care Workers. Hum. Vaccines Immunother..

[3] Ferrari C., Trabucco Aurilio M., Mazza A., Pietroiusti A., Magrini A., Balbi O., Bolcato M., Coppeta L. (2021). Evaluation of Im-munity for Mumps among Vaccinated Medical Students. Vaccines.

[4] Lee J.E., Lee S.O., Kang J.S., Yi J., Kim K.-H. (2019). Investigation of a Mumps Outbreak in a Dental Clinic at a University Hospital. Infect. Chemother..

[5] Marin M., Marlow M., Moore K.L., Patel M. (2018). Recommendation of the Advisory Committee on Immunization Practices for Use of a Third Dose of Mumps Vi-rus-Containing Vaccine in Persons at Increased Risk for Mumps During an Outbreak. MMWR Morb. Mortal. Wkly. Rep..

[6] Kontio M., Jokinen S., Paunio M., Peltola H., Davidkin I. (2012). Waning antibody levels and avidity: Implications for MMR vac-cine-induced protection. J. Infect. Dis..

[7] Eriksen J., Davidkin I., Kafatos G., Andrews N., Barbara C., Cohen D., Duks A., Griskevicius A., Johansen K., Bartha K. (2013). Seroepidemiology of mumps in Europe (1996–2008): Why do outbreaks occur in highly vaccinated populations?. Epidemimology Infect..

[8] Ministry of Health (2023). National Vaccine Prevention Plan 2023–2025, Italy.

[9] CDC Recommendations of the Immunization Practices Advisory Committee Mumps Prevention. 9 June 1989. https://www.cdc.gov/mmwr/preview/mmwrhtml/00001404.htm.

[10] Italian Society of Occupational Medicine (SIML) (2022). The Main Vaccinations for Healthcare Professionals: Operational guidelines by the SIML Permanent Commission “Health Doctors”.

[11] Dilcher M., Barratt K., Douglas J., Strathdee A., Anderson T., Werno A. (2018). Monitoring viral genetic variation as a tool to im-prove molecular diagnosis for mumps virus. J. Clin. Microbiol..

[12] Vygen S., Fischer A., Meurice L., Njoya I.M., Gregoris M., Ndiaye B., Ghenassia A., Poujol I., Stahl J.P., Antona D. (2016). Waning immunity against mumps in vaccinated young adults, France 2013. Eurosurveillance.

[13] Ramanathan R., Voigt E., Kennedy R., Poland G. (2018). Knowledge gaps persist and hinder progress in eliminating mumps. Vaccine.

[14] Rubin S., Kennedy R., Poland G. (2016). Emerging mumps infection. Pediatr. Infect. Dis. J..

[15] Carr M.J., Moss E., Waters A., Dean J., Jin L., Coughlan S., Connell J., Hall W.W., Hassan J. (2010). Molecular epidemiological evaluation of the recent resurgence in mumps virus infections in Ireland. J. Clin. Microbiol..

[16] Nelson G.E., Aguon A., Valencia E., Oliva R., Guerrero M.L., Reyes R., Lizama A., Diras D., Mathew A.M., Camacho E.J.M. (2013). Epidemiology of a mumps outbreak in a highly vaccinated island population and use of a third dose of mea-sles-mumps-rubella vaccine for outbreak control—Guam 2009 to 2010. Pediatr. Infect. Dis. J..

[17] Lee C.Y., Tang R.B., Huang F.Y., Tang H., Huang L.M., Bock H.L. (2002). A new measles mumps rubella (MMR) vaccine: A ran-domized comparative trial for assessing the reactogenicity and immunogenicity of three consecutive production lots and comparison with a widely used MMR vaccine in measles primed children. Int. J. Infect. Dis..

[18] McLean G.S., Fiebelkorn H.Q., Temte A.P., Wallace J.L. (2013). Prevention of measles, rubella, congenital rubella syndrome, and mumps, 2013: Summary recommendations of the Advisory Committee on Immunization Practices (ACIP). MMWR Recom-Mendation Rep..

[19] Wiedermann U., Garner-Spitzer E., Wagner A. (2016). Primary vaccine failure to routine vaccines: Why and what to do?. Hum. Vaccines Immunother..

[20] Barskey A.E., Glasser J.W., LeBaron C.W. (2009). Mumps resurgences in the United States: A historical perspective on unexpected elements. Vaccine.

[21] Dayan G.H., Quinlisk M.P., Parker A.A., Barskey A.E., Harris M.L., Schwartz J.M.H., Hunt K., Finley C.G., Leschinsky D.P., O’Keefe A.L. (2008). Recent resurgence of mumps in the United States. N. Engl. J. Med..

[22] Date A.A., Kyaw M.H., Rue A.M., Julie K., Obrecht L., Krohn T., Rowland J., Rubin S., Safranek T.J., Bellini W.J. (2008). Long-term persistence of mumps antibody after receipt of 2 measles-mumps-rubella (MMR) vaccinations and antibody response after a third MMR vaccination among a uni-versity population. J. Infect. Dis..

[23] Lewnard J.A., Grad Y.H. (2018). Vaccine waning and mumps re-emergence in the United States. Sci. Transl. Med..

[24] Kennedy R.B., Ovsyannikova I.G., Thomas A., Larrabee B.R., Rubin S., Poland G.A. (2019). Differential durability of immune responses to measles and mumps following MMR vaccination. Vaccine.

[25] Hassan J., Dean J., Moss E., Carr M.J., Hall W.W., Connell J. (2012). Seroepidemiology of the recent mumps virus outbreaks in Ireland. J. Clin. Virol..

[26] Rubin S.A., Qi L., Audet S.A., Sullivan B., Carbone K.M., Bellini W.J., Rota P.A., Sirota L., Beeler J., Timmann C. (2008). Antibody induced by immunization with the Jeryl Lynn mumps vaccine strain effectively neutralizes a heterologous wild-type mumps virus associated with a large outbreak. J. Infect. Dis..

[27] Kenny L., O’Kelly E., Connell J., De Gascun C., Hassan J. (2016). Mumps outbreaks in a highly vaccinated population: Investigation of a neutralization titer against the currently circulating wildtype genotype G5 mumps virus. J. Clin. Virol..

[28] LeBaron C.W., Forghani B., Beck C., Brown C., Bi D., Cossen C., Sullivan B.J. (2009). Persistence of mumps antibodies after 2 doses of measles-mumps-rubella vaccine. J. Infect. Dis..

[29] Gothefors L., Bergstrom E., Backman M. (2001). Immunogenicity and reactogenicity of a new measles, mumps and rubella vaccine when administered as a second dose at 12 years of age. Scand. J. Infect. Dis..

[30] Cohen C., White J.M., Savage E.J., Glynn J.R., Choi Y., Andrews N., Brown D., Ramsay M.E. (2007). Vaccine efficacy estimates, mumps epidemic 2004-2005, England. Emerg. Infect. Dis..

[31] Peltola H., Kulkarni P.S., Kapre S.V., Paunio M., Jadhav S.S., Dhere R.M. (2007). Mumps outbreaks in Canada and the United States: Time for new thinking on mumps vaccines. Clin. Infect. Dis..

[32] Shah M., Quinlisk P., Weigel A., Riley J., James L., Patterson J., Hickman C., Rota P.A., Stewart R., Clemmons N. (2018). Mumps outbreak in a highly vaccinated universi-ty-affiliated setting before and after a measles-mumps-rubella vaccination campaign- Iowa, July 2015–May 2016. Clin. -Fectious Dis..

[33] Golwalkar M., Pope B., Stauffer J., Snively A., Clemmons N. (2018). Mumps outbreaks at four universities-Indiana, 2016. Morb. Mortal. Wkly. Rep..

[34] Cardemil C.V., Dahl R.M., James L., Wannemuehler K., Gary H.E., Shah M., Marin M., Riley J., Feikin D.R., Patel M. (2017). Effectiveness of a Third Dose of MMR Vaccine for Mumps Outbreak Control. N. Engl. J. Med..

[35] Dhiman N., Ovsyannikova I.G., Jacobson R.M., Vierkant R.A., Pankratz V.S., Jacobsen S.J., Poland G.A. (2005). Correlates of lymphoproliferative responses to measles, mumps, and rubella (MMR) virus vaccines following MMR-II vaccination in healthy children. Clin. Immunol..

[36] Hanna-Wakim R., Yasukawa L.L., Sung P., Arvin A.M., Gans H.A. (2008). Immune responses to mumps vaccine in adults who were vaccinated in childhood. J. Infect. Dis..

[37] Rubin S.A., Link M.A., Sauder C.J., Zhang C., Ngo L., Rima B.K., Duprex W.P. (2012). Recent mumps outbreaks in vaccinated populations: No evidence of immune escape. J. Virol..

[38] Zipfel C.M., Colizza V., Bansal S. (2021). The missing season: The impacts of the COVID-19 pandemic on influenza. Vaccine.

[39] Chen B., Wang M., Huang X., Xie M., Pan L., Liu H., Liu Z., Zhou P. (2021). hanges in Incidence of Notifiable Infectious Diseases in China Under the Prevention and Control Measures of COVID-19. Front. Public Health.

[40] Chow E.J., Uyeki T.M., Chu H.Y. (2022). The effects of the COVID-19 pandemic on community respiratory virus activity. Nat. Rev. Microbiol..

[41] Smetana J., Chlibek R., Hanovcova I., Sosovickova R., Smetanova L., Polcarova P., Gal P., Dite P. (2018). Serological investigation of mumps antibodies in adults in the Czech Republic and the need for changes to the vaccination strategy. Hum. Vaccines Immunother..

[42] Braeye T., Linina I., De Roy R., Hutse V., Wauters M., Cox P., Mak R. (2014). Mumps increase in Flanders, Belgium, 2012–2013: Results from temporary mandatory notification and a cohort study among university students. Vaccine.

[43] Domínguez A., Torner N., Castilla J., Batalla J., Godoy P., Guevara M., Carnicer D., Caylà J., Rius C., Jansà J.M. (2010). Mumps vaccine effectiveness in highly im-munized populations. Vaccine.

[44] Waugh C.J., Willocks L.J., Templeton K., Stevenson J. (2020). Recurrent outbreaks of mumps in Lothian and the impact of waning immunity. Epidemiol. Infect..

[45] European Centre for Disease Prevention and Control (2024). Measles on the Rise in the EU/EEA: Considerations for Public Health Response.

[46] Iro M.A., Sadarangani M., Goldacre R., Nickless A., Pollard A.J., Goldacre M.J. (2017). 30-year trends in admission rates for en-cephalitis in children in England and effect of improved diagnosis and measles-mumps-rubella vaccination: A population-based observational st. Lancet Infect. Dis..

[47] Connell A.R., Connell J., Leahy T.R., Hassan J. (2020). Mumps Outbreaks in Vaccinated Populations—Is It Time to Re-assess the Clinical Efficacy of Vaccines?. Front. Immunol..

[48] Yung C.-F., Andrews N., Bukasa A., Brown K.E., Ramsay M. (2011). Mumps complications and effects of mumps vaccination, England and Wales, 2002–2006. Emerg. Infect. Dis..

[49] Zamir C.S., Schroeder H., Shoob H., Abramson N., Zentner G. (2015). Characteristics of a large mumps outbreak: Clinical severity, complications and association with vaccination status of mumps outbreak cases. Hum. Vaccines Immunother..

[50] Beleni A.-I., Borgmann S. (2018). Mumps in the Vaccination Age: Global Epidemiology and the Situation in Germany. Int. J. Environ. Res. Public Health.

[51] Rasheed M.A.U., Hickman C.J., McGrew M., Sowers S.B., Mercader S., Hopkins A., Grimes V., Yu T., Wrammert J., Mulligan M.J. (2019). Decreased humoral immunity to mumps in young adults immunized with MMR vaccine in childhood. Proc. Natl. Acad. Sci. USA.

[52] Flanagan K.L., Fink A.L., Plebanski M., Klein S.L. (2017). Sex and Gender Differences in the Outcomes of Vaccination over the Life Course. Annu. Rev. Cell Dev. Biol..

[53] Klein S.L., Jedlicka A., Pekosz A. (2010). The Xs and Y of immune responses to viral vaccines. Lancet Infect. Dis..

[54] Choi K.M. (2010). Reemergence of mumps. Korean J. Pediatr..

[55] Ryu J.-U., Kim E.-K., Youn Y.-S., Rhim J.-W., Lee K.-Y. (2014). Outbreaks of mumps: An observational study over two decades in a single hospital in Korea. Korean J. Pediatr..

[56] Italian Parliament (2017). Italian Legislative Decree 73/2017.

[57] ISS Epicentro Le Vaccinazioni in Italia. https://www.epicentro.iss.it/vaccini/dati_Ita#parotite.

[58] Terada K., Itoh Y., Wakabayashi T., Teranishi H., Akaike H., Ogita S., Ouchi K. (2015). Rubella specific cell-mediated and humoral immunity following vaccination in college students with low antibody titers. Vaccine.

[59] Dayan G.H., Rubin S. (2008). Mumps outbreaks in vaccinated populations: Are available mumps vaccines effective enough to prevent outbreaks?. Clin. Infect. Dis..

[60] Amanna I.J., Carlson N.E., Slifka M.K. (2007). Duration of humoral immunity to common viral and vaccine antigens. N. Engl. J. Med..

[61] Paris C., Bénézit F., Geslin M., Polard E., Baldeyrou M., Turmel V., Tadié É., Garlantezec R., Tattevin P. (2021). COVID-19 vaccine hesitancy among healthcare workers. Infect. Dis. Now.

[62] Aurilio M.T., Mennini F.S., Gazzillo S., Massini L., Bolcato M., Feola A., Ferrari C., Coppeta L. (2021). Intention to Be Vaccinated for COVID-19 among Italian Nurses during the Pandemic. Vaccines.

[63] Coppeta L., Morucci L., Pietroiusti A., Magrini A. (2019). Cost-effectiveness of workplace vaccination against measles. Hum. Vaccines Immunother..

